# Progestin-based pharmacotherapy in fertility preservation in early endometrial cancer

**DOI:** 10.3389/fonc.2024.1487008

**Published:** 2024-11-11

**Authors:** Zhendong Qin, Di Zhang, Guangming Cao, Hua Li

**Affiliations:** Beijing Chaoyang Hospital, Capital Medical University, Beijing, China

**Keywords:** progestin, endometrial cancer, conservative treatment, retention of fertility, drug therapy

## Abstract

Endometrial cancer is a common tumor of the female reproductive system. In recent years, as the age of onset of the disease has gradually become younger, this has caused distress to some young patients with reproductive needs, and the active search for methods of preserving reproductive function has gradually attracted attention. In this paper, we will systematize the current status of progestin-based pharmacotherapy in combination with other drug therapies in the conservative management of early-stage endometrial cancer. With the expectation of providing a reference for the treatment of early stage endometrial cancer patients in China and for the in-depth development of related research in this field.

## 1 Introduction

Endometrial cancer (EC) is one of the three most common malignant tumors in gynecology (1). And its incidence rate in China is only second to that of cervical cancer. However, due to lifestyle changes, its incidence rate is rising, and with the popularization of cervical cancer screening and prevention, endometrial cancer has surpassed cervical cancer in developed countries as the most common malignant tumor of the female reproductive tract, posing a serious threat to women’s health (2–4). Risk factors for endometrial cancer include advanced age, high Bodymass index (BMI) endogenous or exogenous estrogen exposure, early menarche, late menopause, infertility, metabolic syndrome, and Lynch syndrome, whereas normal BMI, multiple births, and use of oral contraceptives are protective factors for EC (5–7). With the comprehensive and high-speed economic and social development, late marriage, late childbearing and fewer children has become an unavoidable social trend under the economic and social development, coupled with high oil, high-fat and high-sugar dietary habits, so that the incidence of EC is increasing year by year and the age of onset of the disease is becoming younger (8, 9).

EC is divided into two types: type I and type II (10). Type I EC, also known as “estrogen-dependent”, includes G1 and G2 endometrioid adenocarcinomas (11), which account for more than 80% of all EC, and may be related to the long-term effects of estrogen without progestin antagonism. The patients are usually young with a history of irregular vaginal bleeding and develop from atypical endometrial hyperplasia (AH) (12). Type II EC, also known as “non-estrogen-dependent”, includes G3 endometrioid adenocarcinoma, plasma carcinoma, clear cell carcinoma, carcinosarcoma, and undifferentiated carcinoma, etc (12). There is no clear relationship between type II EC and estrogen, and it is most common in older, thin women, who do not have endometrial thickening or bleeding symptoms (13). Its diagnosis by crude screening methods of abnormal bleeding symptoms or endothelial thickness is more likely to be missed, and the worse prognosis of type I EC compared with type I EC is strongly related to the inability to detect it early (13, 14). Currently, the diagnosis of endometrial cancer relies on surgical procedures such as diagnostic curettage referred to as “diagnostic scraping”, segmental diagnostic scraping and hysteroscopy to obtain endometrial tissue for histopathological examination (15).

In conclusion, although total hysterectomy combined with bilateral tubo-ovariectomy is the gold standard in the treatment of endometrial cancer, conservative treatment is necessary in young patients with endometrioid tissue type, International Federation of Gynecology and Obstetrics (FIGO) tumor grade 1, no extra-uterine metastases, and no lymphovascular interstitial invasion, myometrial or cervical invasion (16, 17).

## 2 The need to preserve fertility

Currently, the recommended treatment for endometrial cancer is hysterectomy combined with bilateral tubo-ovariectomy with or without lymph node dissection with pelvic cleansing, sometimes combined with adjuvant chemotherapy or radiotherapy (16, 18, 19). Although this is a very effective method, with a 5-year survival rate of 93%, it also results in permanent loss of fertility and affects the patient’s quality of life and psychological well-being, which is unacceptable to young women who wish to maintain their fertility (20–22). Although endometrial cancer and its precancerous lesions are most commonly seen in peri-menopausal and postmenopausal women, some patients are younger, and the proportion of young patients is increasing, showing a global trend towards younger age. Statistically, about 14% of new cases of endometrial cancer are in women of childbearing age less than 40 years old, and about 75% of them have a desire to preserve the uterus or reproductive function (23, 24). About 80% of young endometrial cancer patients are staged as type I. It often develops from atypical hyperplasia of the endometrium, its precancerous lesion, and is usually well differentiated and the tumor is confined to the endometrium, and most of them are estrogen-dependent with a better prognosis. For stage Ia patients, the risk of pelvic lymph node metastasis is only 1% to 5%, and the risk of ovarian metastasis is only 1%. Based on the above characteristics, for young patients with early endometrial cancer and precancerous lesions who have fertility requirements, fertility preservation treatment can be considered (25).

Since Kistner’s team (26) first reported progestin therapy for endometrial cancer in 1959, fertility preservation therapy forendometrial cancer has gained increasing attention. Currently, the clinical effectiveness of conservative treatment with progestin can reach about 70% to 80%, however, even with the help of assisted reproductive technology, the pregnancy rate is still less than 40% (27). In recent years, in response to the aging of the population, the delay in the age of childbearing and the decline in the fertility rate, the State has opened up the three-child policy (28). Therefore, how to improve the success rate of pregnancy in such patients is an urgent clinical challenge.

## 3 The indications and contraindications for fertility preservation

Currently, there is no uniform view on the indications for fertility preservation therapy, but most of them believe that under the safety of oncologic therapy, patients with endometrial cancer who meet the following indications can have their fertility preserved: ① Age <40 years old and strong desire to preserve fertility; ② Endometrioid adenocarcinoma (type I), stage 1a; ③ Pathological diagnosis of highly to moderately differentiated; ④ Immunohistochemical staining suggestive of progesterone receptor positivity; ⑤ Tumor is limited to the body of the uterus, with no myometrial infiltration and no extra-uterine metastases (29). For those with the following risk factors, conservative treatment is not suitable, and surgery is recommended according to the guidelines: ① non-endometrioid adenocarcinoma; ② immunohistochemistry suggests that the progesterone receptor is negative; ③ pathology suggests that it is poorly differentiated; ④ found that the ovary is involved or combined with ovarian cancer; ⑤ tumor invasion of the deep muscle layer or cervix; ⑥ combined with liver and kidney function damage, cardiopulmonary function, coagulation disorders and other systemic diseases ⑦ Combined with severe uterine malformation or endometrial tuberculosis; ⑧ Poor follow-up conditions and poor compliance.

## 4 Drug therapy for fertility preservation

### 4.1 Oral progestogen

At present, the methods of conservative treatment of endometrial atypical hyperplasia and early endometrial cancer have not been standardized. At present, the most commonly used and effective ones are mainly high-efficiency progestins. These include medroxyprogesterone acetate (MPA), medroxyprogesterone acetate (MA), levonorgestrel intrauterine device (LNG-IUD), progesterone, oral contraceptives, and progesterone caproate (28, 30). Oral progestogen treatment is the mainstay, most commonly Medroxyprogesterone acetate (MPA) 500-600mg once daily or Megestrol acetate (MA) 160-480mg once daily (31). Progestogen binding to the progesterone receptor (PR) delays DNA and RNA replication and decreases estrogen receptor (ER) expression, which in turn reduces endometrial cancer cell proliferation and promotes cell differentiation (32). The median duration of high-dose progestin treatment for endometrial atypical hyperplasia and early endometrial cancer CR is 6 months; if complete remission is not achieved after 9-12 months of treatment, it is recommended to change to surgical treatment, or the duration of the medication can be extended according to the therapeutic effect, but generally not more than 1 year (33); For persistent or progressive lesions, the treatment should be changed to surgery (34). Typically, patients who achieve CR with conservative treatment are advised to become pregnant as early as possible, and assisted reproduction is generally recommended as soon as possible, through which the risk of recurrence can be reduced (35).

In conclusion, compared with curettage, high-dose progestin oral therapy effectively avoids instrumental damage to the patient’s uterus, as well as the risk of intraoperative metastasis of cancer cells, and reduces the risk of recurrence while protecting the patient’s function. At the same time, progestogens mainly act on the patient’s endometrium, stimulating the proliferation and secretion of the endometrium, increasing the amount of negative feedback to the hypothalamus, thereby effectively inhibiting the production and release of luteinizing hormone in the anterior pituitary gland, inhibiting ovarian ovulation, and realizing treatment.

### 4.2 Combined LNG-IUD

However, there are some problems associated with oral high-dose progestins. On the one hand, it can lead to common side effects such as breast pain, breast spillage, vaginal bleeding, weight gain, headaches, and mood changes, and on the other hand, since oral medications need to be taken over a long period of time, they require good self-consciousness and compliance on the part of the patient. To overcome this problem, intrauterine levonorgestrel intrauterine device (LNG-IUD) has also been used to treat early endometrial cancer and precancerous lesions (36).

Progestin-containing intrauterine devices (IUDs) offer an attractive method of delivering progestin to the localized endometrium and preventing atypical endometrial hyperplasia in patients with early-stage endometrial cancer who are being treated conservatively (37, 38). The LNG-IUD is a localized, sustained-release system of progestin, marketed and manufactured by Bayer AG, Germany (39–41). Currently commercially available progestin-containing intrauterine devices (IUDs) are categorized as Mirena, Jaydess, and Kyleena, depending on the dosage they contain (42, 43). In 1990, an intrauterine device (IUD) containing levonorgestrel (L-norgestrel) was introduced to the market under the name Mirena, which provides effective release for up to 5 years (44). The device releases up to 20 μg of levonorgestrel per day (average 14ug/24h) directly into the uterine cavity, creating a high concentration of progestin in the uterine cavity, strongly inhibiting atypical endometrial hyperplasia, generating high endometrial concentrations and low blood levels, with only minor adverse effects on body metabolism (45, 46). In 2013, a smaller IUD, marketed as Skyla or Jaydess, was introduced that contains 13.5 mg of levonorgestrel and releases approximately 10 μg of levonorgestrel per day, tapering down to 5 μg/d, which provides an effective release for 3 years (47). In 2016, Bayer of Germany received approval from the U.S. Food and Drug Administration (FAD) for its new IUD, Kyleena, which is the third IUD after Mirena and Jaydess. Each Kyleena contains 19.5 mg of the progestin hormone levonorgestrel, which provides effective release for up to five years after use (48).

Jaydess/Skyla and Kyleena are “low-dose” levonorgestrel-releasing intrauterine systems (LNG-IUS) compared to Mirena (49). Jaydess/Skyla and Kyleena have lower LNG content and smaller sizes, which make placement easier and less painful. placement while maintaining similar efficacy (43, 49, 50). The low-dose LNG system also reduces the incidence of overall adverse reactions, including obesity, metabolic disturbances, and other side effects of progestin overdose (51–53). However, the Mannheimer’s Ring, as a foreign body in the uterus, can still produce an inflammatory response, and patients may often present with irregular vaginal bleeding, lower abdominal pain, infections, and uterine perforation, and will need to go to the operating room to have it removed prior to a planned pregnancy (54).

### 4.3 Combined hysteroscopic treatment

Conservative treatment of early endometrial cancer by scraping to achieve removal of the cancer was first reported in the 1960s (55). With the rapid development of medical science and technology and the rapid maturation of gynecological endoscopy technology, hysteroscopy has been rapidly applied to all aspects of the diagnosis and treatment of gynecological diseases in recent decades.Gonthier C proposes a new idea of hysteroscopic lesion electrosurgery combined with high-efficiency progestin for the treatment of early EC with preservation of reproductive function (56). Hysteroscopic Focal Electrodessication (HFE) is the procedure of removing the lesion in the uterine cavity and its surrounding tissues under the direct vision of the hysteroscope to minimize the cancerous foci and protect other normal tissues and endothelial lining from being damaged, so as to achieve the optimization of the cure of the disease and the protection of the reproductive function (57, 58). The resected tissues are sent for pathologic examination, and if they meet the indications of preserving reproductive function, they are combined with high-efficiency progestin for postoperative maintenance therapy, and progestin acts on the endometrium to further inhibit and eliminate the remaining cancer foci (59).

Crosbie et al. concluded that hysteroscopic lesion electrosurgery combined with high-efficiency progestins not only reduces the tumor load of subsequent drug therapy but also effectively reduces the application of progestins, decreases the side effects of progestins, improves the efficacy of treatment, and shortens the treatment period at the same time (20). Study demonstrates high complete remission rates in patients with fertility-preserving early-stage endometrial cancer treated with hysteroscopic lesion electrodesiccation and subsequent high-potency progestin therapy (60, 61). However, the application of hysteroscopy is easy to cause damage to the endometrium, after the operation, the patient may be complicated by uterine deformity, endometrial thinning, endometrial inflammation of the uterine cavity adhesions and other changes that lead to a decrease in the endometrial tolerance, which is not conducive to embryo implantation, and is prone to miscarriage, preterm labor, and so on (62, 63). In addition, Kandoth et al. suggested that the application of hysteroscopy may cause intraperitoneal dissemination of cancer cells, but no study has shown that the prognosis of patients is affected by it (62, 64).

In conclusion, whether hysteroscopic lesion electrodesiccation has a better oncologic prognosis in the management of patients with EC, and the impact of postoperative complications such as the occurrence of uterine adhesions on pregnancy outcomes is unclear, and the controversial nature of its application suggests that a large number of prospective studies with large samples are still needed for further exploration.

### 4.4 Combined synthetic gonadotropin-releasing hormone analogs

GnRH is a peptide hormone secreted by the hypothalamus that stimulates the pituitary gland to synthesize gonadotropins, thereby promoting the secretion of sex hormones by the ovaries (65). Gonadotropin-releasing hormone agonist (GnRH agonist, GnRH-a) is a synthetic derivative of GnRH-a,by altering amino acids 6 and 10 of GnRH-a, the newly produced skin chain is structurally stabilized, the half-life is extended (1-6h), and the binding capacity to the corresponding receptor is increased 100-200-fold, which enables GnRH-a to inhibit the secretion of pituitary gonadotropins (66). The mechanism of action of GnRH-a in the treatment of EC is mainly desensitization, i.e., when the patient first uses the drug for 7-14 days, it will make the pituitary gonadotropin transiently elevated, and if it is used continuously, the pituitary function will be inhibited to appear down-regulation, which will result in a significant decrease in the levels of serum follicular spiking (folliclestimulating hormone (FSH), luteinizing hormone (LH), and ovarian sex hormones, and thus achieve the effect of inhibiting endometrial hyperplasia. Luteinizing hormone (LH), ovarian sex hormone levels decreased significantly, thus achieving the effect of inhibiting endothelial hyperplasia (65, 67–69); In addition, GnRH-a can directly bind to GnRH receptors on tumor cells, interfering with mitotic signaling, inducing a decrease in c-fos expression of oncogenes, and inhibiting tumor cell proliferation, thus inhibiting tumor growth (70, 71). Tang et al. found that GnRH-a inhibited the proliferation of EC cells in *in vitro* experiments (72). Pashov et al. reported 9 patients with early EC who were given 9 consecutive injections of GnRH-a and placed on LNG-IUS for at least 1 year after the 3rd injection, and achieved a favorable outcome (73). Sallam et al. showed that the use of GnRH-a for 3-6 months resulted in a 4-fold increase in the clinical pregnancy rate, which may improve the conception rate of patients while treating endothelial pathologies (74). Since its reversal rate can reach 35%, it has been proposed for the treatment of endometrial cancer due to atypical endometrial hyperplasia.

Compared with oral progestogen, this drug is injected once a month, avoiding the disadvantage of progestogen needing to be taken orally every day, which can improve patient compliance, and the chance of liver and kidney function damage is small, but the price is relatively high.The main adverse effects are menopausal symptoms associated with reduced estrogen and progestogen levels, which can be relieved by oral medication such as Livastigmine.

### 4.5 Combined metformin

In patients with polycystic ovary syndrome (PCOS), the body is in a progesterone-deficient state and the endometrium is in a state of estrogen-mediated proliferation for a long period of time due to prolonged anovulation or reduced ovulation, which may be an important reason for the high incidence of endometrial cancer in patients with polycystic ovary syndrome (75–77). Most clinical features of patients with polycystic ovary syndrome are characterized by hyperandrogenism, scanty or amenorrhea, anovulatory infertility, hirsutism, insulin resistance, and type II diabetes mellitus (78–80). Meanwhile, studies have shown that obesity, diabetes, and insulin resistance are risk factors for the development and progression of endometrial cancer (81, 82). Metformin, an oral hypoglycemic agent of the biguanide class, is now widely used in the treatment of type II diabetes mellitus to improve insulin resistance (83). Therefore, metformin can also be used in the conservative treatment of patients with early-stage endometrial cancer due to PCOS.

The study showed that the combination of metformin and oral progestins not only modified the insulin resistance status, but also that the combination of metformin and oral progestins remained effective in patients with atypical endometrial hyperplasia combined with polycystic ovary syndrome, even if oral progestins alone failed to treat the disease (84). Meanwhile, Shan et al (85). reported 2 cases (25%) in which combined metformin administration still failed to reverse endometrial histology to normal. However, the mechanism of action of metformin is very complex, and the more clearly studied mechanism is the activation of mitogen-activated protein kinase (AMPK), which in turn inhibits tumor suppressor genes such as PTEN/AKT and TSC2/TSC1 (86). The end result of these effects is the indirect inhibition of mTOR, a central cell growth activator, through the PI3K/Akt pathway (86–89), and through the induction of caspases 3/7, 8, 9 and proteins that regulate autophagy and cell death (e.g. bcl2 and beclin 1) leading to apoptosis (90–92). In endometrial cancer, metformin decreases cell viability and proliferation in a human endometrial cancer cell line (Ishikawa cells) through activation of pAMPK, induction of beclin 1, down-regulation of IGF, and increased autophagy and apoptosis (92).

### 4.6 Combined tamoxifen

Estrogen overstimulation is the main cause of EC. Progesterone antagonizes estrogenic activity by competitively blocking estrogen receptors and is the basic treatment for EC (93). However, prolonged progesterone therapy leads to decreased regulation of cytoplasmic and nuclear receptors, as well as lack of functional progesterone receptor expression in some patients, which in turn affects the anti-endothelial cell proliferation and differentiation effects of progesterone.

Tamoxifen (TAM) belongs to the group of estrogen receptor modulators and acts as both an antagonist and an agonist of the estrogen receptor, depending on the target tissue. As early as 1985, Killackey et al. suggested a possible link between tamoxifen use and the development of endometrial cancer (94). This was confirmed by a series of later studies that showed tamoxifen users had a 1.5 to 6.9 times increased risk of EC (95). However, the risk of tamoxifen-associated EC was not related to the daily dose of the medication, but rather to the duration of the medication and cumulative dose (96–99). Postmenopausal and elderly patients have a higher risk of EC than premenopausal and younger women, and it increases with weight gain (95, 96, 100). Therefore, in young patients with endometrial cancer, tamoxifen alone or in combination with progesterone can be used in the short term as an effective adjuvant therapy with low toxicity (101–103). Because TAM has a composition structure similar to that of estrogen, it can competitively bind with estrogen receptor in the body after entering the human body, thus inhibiting estrogen from binding to it, and at the same time it can stimulate the excitation of progesterone receptor and improve its activity, effectively regulating the estrogen and progestogen levels in the patient’s body, inhibiting the growth and proliferation of tumor cells, and further enhancing the clinical efficacy of the treatment after being used in conjunction with MA.

As the preferred second-line hormone therapy after first-line progestogen therapy, especially for endometrial tumors and estrogen receptor-positive patients, it is effective in 10%-53% of cases (102). Thus, tamoxifen is a dual drug with regard to EC: on the one hand, it increases the risk of the development of this disease, and on the other hand, it acts as a remedy that somehow slows down the development of the tumor. Thus, tamoxifen is a dual drug with regard to EC: on the one hand, it increases the risk of the development of this disease, and on the other hand, it acts as a remedy that somehow slows down the development of the tumor.

## 5 Assessment of outcomes after fertility preservation treatment

Since the earliest progestin effect on endometrial cancer cells occurs at 10 weeks after the start of treatment, the first evaluation of the efficacy of conservative treatment should be at week 12. It is recommended that patients with endometrial cancer undergo a follow-up examination every 3 months after starting progestin therapy, which includes gynecological examination, MRI or vaginal ultrasonography, diagnostic curettage and hysteroscopy (104, 105). Efficacy was graded according to the results of the tests, which were categorized as complete remission, partial remission, no change, and disease progression, and if any suspicious signs or symptoms appeared during the treatment period, they should be seen immediately.

For patients in complete remission with no history of secondary or primary infertility, conception can be attempted after discontinuation of the drug; or after 9 months of consolidation of the drug before attempting conception. Since young patients with endometrial cancer often have other factors that affect their fertility, such as obesity, long-term anovulation and polycystic ovary syndrome, the pregnancy rate of spontaneous conception is still low (106). If attempts to conceive naturally for 3 months are unsuccessful, the use of appropriate assisted reproductive technologies is recommended to prevent the tumor from recurring and missing the opportunity to have children. In patients with a proven history of infertility or anovulation, ovulation induction should be initiated as soon as complete remission is indicated. However, there is no evidence on whether assisted reproductive technologies such as ovulation induction increase the risk of endometrial cancer recurrence.

## 6 Conclusion

In conclusion, there are many conservative treatments for early stage endometrial cancer. Progestogen is the most commonly used and effective method. It can affect endometrial cancer cells through multiple signaling pathways and multiple molecular targets, such as the CACNA2D3 gene, Wnt/β-catenin, PI3K/Akt, LncRNANEAT1/miR-146b-5p, PDCD4, and TGF-β/Smads, to influence endometrial cancer cell proliferation, apoptosis, invasion and metastasis, thus exerting anti-tumor effects (107–111). Hysteroscopic endometrial electrosurgery combined with progestogen in the treatment of endometrial cancer has a favorable prognosis while preserving fertility (112). In addition, the combined application of multiple methods will realize good results. As far as the course of treatment is concerned, MPA for 6 months; MA for 3-14 months;GnRHa+LNG-IUD, for 1 year; hysteroscopic electrosurgery+LNG-IUD,for 1 year;andhysteroscopic electrosurgery+MA,160mg/day, for 6 months (113, 114). However, there is a lack of side-by-side comparisons of various therapies to develop an individualized formulation, and further research is needed on starting dose, treatment period, efficacy assessment, follow-up time, timing of pregnancy, and mode of pregnancy.

In summary, for endometrioid adenocarcinoma patients under 40 years of age with a strong desire to preserve fertility, endometrioid adenocarcinoma (type I), stage 1a, G1; MRI (preferred) or transvaginal ultrasonography showing lesions confined to the endometrium (normal CA125 level, pathologic diagnosis of highly to moderately differentiated and ER-positive, and tumors confined to the body of the uterus) and no extrauterine metastases, conservative treatment is feasible. However, when natural conception is unsuccessful for 3 months in patients with preserved reproductive function, they should be supported by assisted human reproductive technology (ART), and the drugs used in the ART process do not increase the risk of EC recurrence. Therefore, ART can be an important way to assist patients with early-stage EC and EAH to complete their reproductive function after successful conservative treatment (115–117). Conservative treatment to preserve fertility is feasible, and treatment options vary depending on the patient’s condition. However, preservation of reproductive function is only temporary. For patients after successful delivery, postpartum surgery for full staging of endometrial cancer is still recommended, and the decision to preserve or not to preserve the double adnexa should be made after rigorous evaluation of the patient’s condition (118).

In conclusion, the treatment of young EC patients with preservation of fertility is now a more mature diagnostic and therapeutic protocol with good oncologic and pregnancy outcomes after decades of experience at home and abroad. Identification and active intervention of factors influencing outcomes can help to improve the efficacy of fertility preservation therapy. The selection of individualized treatment plans based on patients’ individual characteristics is a direction for future research. In addition, the exploration of fertility preservation treatment for patients with special conditions requires in-depth observation and research. With the help of advanced molecular pathology technology, we should also pay attention to the direction of more accurate screening and optimization of patients who are suitable for fertility preservation.
